# Projecting kelp (*Ecklonia radiata*) gametophyte thermal adaptation and persistence under climate change

**DOI:** 10.1093/aob/mcad132

**Published:** 2023-09-04

**Authors:** R J Veenhof, C Champion, S A Dworjanyn, J Schwoerbel, W Visch, M A Coleman

**Affiliations:** National Marine Science Centre, Faculty of Science and Engineering, Southern Cross University, Coffs Harbour, NSW, Australia; National Marine Science Centre, Faculty of Science and Engineering, Southern Cross University, Coffs Harbour, NSW, Australia; Fisheries Research, NSW Department of Primary Industries, National Marine Science Centre, Coffs Harbour, NSW, Australia; National Marine Science Centre, Faculty of Science and Engineering, Southern Cross University, Coffs Harbour, NSW, Australia; Institute for Marine and Antarctic Studies, University of Tasmania, Hobart, Tasmania, Australia; Institute for Marine and Antarctic Studies, University of Tasmania, Hobart, Tasmania, Australia; National Marine Science Centre, Faculty of Science and Engineering, Southern Cross University, Coffs Harbour, NSW, Australia; Fisheries Research, NSW Department of Primary Industries, National Marine Science Centre, Coffs Harbour, NSW, Australia

**Keywords:** Climate change, *Ecklonia radiata*, gametophytes, generalized additive modelling, genetic clusters, kelp forests, thermal adaptation, ocean warming, spatial projections, range edge, thermal resilience

## Abstract

**Background and aims:**

Kelp forests underpin temperate marine ecosystems but are declining due to ocean warming, causing loss of associated ecosystem services. Projections suggest significant future decline but often only consider the persistence of adult sporophytes. Kelps have a biphasic life cycle, and the haploid gametophyte can be more thermally tolerant than the sporophyte. Therefore, projections may be altered when considering the thermal tolerance of gametophytes.

**Methods:**

We undertook thermal tolerance experiments to quantify the effect of temperature on gametophyte survival, relative growth rate (RGR) and sex ratio for three genetically distinct populations of *Ecklonia radiata* gametophytes from comparatively high, mid- and low latitudes (43°, 33° and 30°S). We then used these data to project the likely consequences of climate-induced thermal change on gametophyte persistence and performance across its eastern Australian range, using generalized additive and linear models.

**Key results:**

All populations were adapted to local temperatures and their thermal maximum was 2–3 °C above current maximum *in situ* temperatures. The lowest latitude population was most thermally tolerant (~70 % survival up to 27 °C), while survival and RGR decreased beyond 25.5 and 20.5 °C for the mid- and low-latitude populations, respectively. Sex ratios were skewed towards females with increased temperature in the low- and high-latitude populations. Spatially explicit model projections under future ocean warming (2050-centred) revealed a minimal decline in survival (0–30 %) across populations, relative to present-day predictions. RGRs were also projected to decline minimally (0–2 % d^−1^).

**Conclusions:**

Our results contrast with projections for the sporophyte stage of *E. radiata*, which suggest a 257-km range contraction concurrent with loss of the low-latitude population by 2100. Thermal adaptation in *E. radiata* gametophytes suggests this life stage is likely resilient to future ocean warming and is unlikely to be a bottleneck for the future persistence of kelp.

## INTRODUCTION

Global climate change is increasingly affecting marine ecosystems (Harley *et al.*, 2006; Doney *et al.*, 2011; Gissi *et al.*, 2021) with rising ocean temperatures driving profound changes to the distribution, ecological structure and reproductive phenology of subtidal marine communities (Sorte *et al.*, 2010; Poloczanska *et al.*, 2013; Boyce *et al.*, 2015). As all marine species have a thermal niche in which they thrive (Tittensor *et al.*, 2010; Stuart-Smith *et al.*, 2017), increasing temperatures are shifting the distribution of species poleward (Sorte *et al.*, 2010; Bates *et al.*, 2014; Chaudhary *et al.*, 2021) and causing local extinctions at trailing range edges (Nicastro *et al.*, 2013; Fredston-Hermann *et al.*, 2020; Pinsky *et al.*, 2020). The impacts of increased temperature may vary across species’ life stages and spatial distribution (Pankhurst and Munday, 2011; de Bettignies *et al.*, 2018), making it vital to capture the thermal response of different life stages and populations to project distributional changes (Foden *et al.*, 2019; Twiname *et al.*, 2020).

Kelp forests are among the coastal marine ecosystems most impacted by global climate change (Bartsch *et al.*, 2012; Harley *et al.*, 2012; Wernberg *et al.*, 2019*b*) with losses of biomass, abundance and range as well as local extinctions documented in North America and Canada (Filbee-Dexter *et al.*, 2016; Berry *et al.*, 2021), California and South America (Schiel *et al.*, 2004; Arafeh-Dalmau *et al.*, 2019), Australia and New Zealand (Johnson *et al.*, 2011; Wernberg *et al.*, 2016; Thomsen *et al.*, 2019) and Europe (Moy and Christie, 2012; Voerman *et al.*, 2013). Kelps are foundation species that provide habitat and vast ecosystem services in temperate marine environments (Dayton, 1985; Steneck *et al.*, 2002), estimated to value up to 684 billion USD annually (Eger *et al.*, 2023). The loss of these foundation species signifies a loss in overall marine biodiversity (Castorani *et al.*, 2018; Gabara *et al.*, 2021; Pessarrodona *et al.*, 2021) and fragmentation of kelp habitat reduces recruitment, triggering further kelp decline (Layton *et al.*, 2020; Reeves *et al.*, 2022).

To enable proactive management and conservation of kelp forests globally, predictive modelling has been used to elucidate where kelp is likely to be lost (Assis *et al.*, 2018; Martínez *et al.*, 2018; Sudo *et al.*, 2020) and to identify areas of persistence in the future (Bekkby and Moy, 2011; Franco *et al.*, 2018; Davis *et al.*, 2021, 2022). Range contractions are common along coastlines affected by ocean warming and can be especially severe where there is no suitable habitat for poleward range expansion. For example, the distribution of sporophyte populations of the main canopy-forming kelp in Australia, *Ecklonia radiata*, are projected to decline by 71 and 49 % under RPC 6.0 and 2.6 emission scenarios, respectively (Martínez *et al.*, 2018). More nuanced estimates show a 257-km range contraction, a shift to deep cooler water and 30 % loss of biomass on Australia’s east coast (Davis *et al.*, 2022). Similarly, suitable habitat for kelp forests in Japan is projected to decline between 50 and 100 % resulting in extinction of six out of 11 native kelp species (Sudo *et al.*, 2020). A common shortcoming of such models used to predict future kelp forest distributions, however, is the consideration of only the macroscopic sporophyte stage of kelp. Despite its importance in kelp forest persistence, the survival and performance of the microscopic gametophyte stage is rarely considered.

Kelps are characterized by a biphasic life history, where the adult diploid sporophyte alternates with the microscopic haploid gametophyte (Pedersen, 1981). The gametophyte stage has been considerably less researched than the sporophyte stage, but is integral for kelp forest persistence (Veenhof *et al.*, 2022*a*) as the stage through which regeneration occurs is pivotal in understanding species reactions to climate change (Parmesan and Hanley, 2015). The complex life history of kelps makes predicting distributional changes convoluted as two separate life stages are to be considered (Murphy *et al.*, 2016, 2017). Most projections of distributional change are based on records of sporophyte presence in the field (i.e. Martínez *et al.*, 2018). Some authors have included theoretical model parameters accounting for dispersal and delayed development of microscopic stages (Assis *et al.*, 2017) or physiological thresholds of the microscopic phase based on the literature (Murphy *et al.*, 2016, 2017). Few authors have tried to experimentally test limits of microscopic life stages and integrate them in models derived from mechanistic relationships (but see Capdevila *et al.*, 2019). Mechanistic relationships derived from experimental data on species’ physiological thresholds have the potential for highly accurate projections and the microscopic life stage of kelp is well suited for this method (Kearney and Porter, 2009; Twiname *et al.*, 2020).

Predictive models based on gametophyte physiology could supplement existing models projecting sporophyte decline, together presenting a holistic view of kelp distributional changes and where critical bottlenecks might lie. Gametophyte temperature thresholds can be higher than those of sporophytes (Bartsch *et al.*, 2013; Mohring *et al.*, 2014; Martins *et al.*, 2017; Becheler *et al.*, 2022), allowing gametophytes to persist through stressful conditions (Barradas *et al.*, 2011; Carney *et al.*, 2013). For example, the known thermal tolerance of *E. radiata* gametophytes ranges between 1 and 28 °C (tom Dieck, 1993; Mabin *et al.*, 2013; Mohring *et al.*, 2014), compared to temperature thresholds of 8 and 25 °C for sporophyte populations (Wernberg *et al.*, 2019*a*). Gametophyte models are likely to present altered projections of decline if gametophytes can survive more extreme temperatures during periods of thermal stress.

Kelps can also display local thermal adaptation throughout their range which, if incorporated into model projections, would likely alter outcomes. Gametophyte populations from warmer edges of their distribution can have a higher thermal tolerance (Oppliger *et al.*, 2012; Mohring *et al.*, 2014; Martins *et al.*, 2020; Liesner *et al.*, 2022), which may be underpinned by both plasticity and heritable genetic variation across a species’ range (King *et al.*, 2019; Vranken *et al.*, 2021; Wood *et al.*, 2021) and so the warmer, trailing edge may respond differently to climate change (Hampe and Petit, 2005; Mota *et al.*, 2018). To accurately predict changes at trailing edges, rather than use a single thermal envelope as a predictor, spatial variation in a species’ thermal tolerance underlaid by genetic variation should be captured in species distribution models (Russell *et al.*, 2012).

Models that explicitly quantify the drivers of early kelp life stages using laboratory-derived data are vital to better understand how kelp forests will cope in the face of climate change (Assis *et al.*, 2017; Twiname *et al.*, 2020). Here we use thermal experiments to develop such models for gametophytes of the main canopy-forming species in Australia, *E. radiata,* sampled across ~1500 km of latitude. First, we identify the specific thermal tolerance of genetically distinct populations of *E. radiata* gametophytes across its range along eastern Australia. Second, we use these data to construct spatially explicit models of gametophyte survival, relative growth rate (RGR) and sex ratio that account for differential responses of thermally adjusted gametophytes. Third, we project these responses under historical and future ocean conditions and compare the change in gametophyte response, and fourth, we compare outcomes of the gametophyte projections to projected changes to sporophyte distributions to create a holistic view of future persistence of *E. radiata* in eastern Australia.

## MATERIALS AND METHODS

### Study species

*Ecklonia radiata* gametophytes were sourced at three different populations (two or three sites within each population) off the east coast of Australia based on distinct genetic clusters (AJP Minne *et al.*, in prep.; Coleman *et al.*, 2011*b*). Approximately ten fertile plants were collected from each site and used to create stock cultures from (1) Charlesworth Bay (30°27ʹS, 153°14ʹʹE), Diggers Beach (30°28ʹS, 153°15ʹE) and Sawtell (30°37ʹS, 153°08ʹE) on 16 July 2021 (population Coffs Harbour), (2) in Bateau Bay (33°23ʹS, 151°29ʹE) and Toowoon Point (33°21ʹS, 151°30ʹE) on the 5 June 2022 (population Sydney) and (3) in Fortescue Bay (43°13ʹS, 147°97ʹE), Apex Point (43°06ʹS, 147°43ʹE) and Coal Point (43°33ʹS, 147°32ʹE) on 13, 11 and 15 August 2020, respectively (population Tasmania). These populations were chosen to represent a comparatively low-latitude (Coffs Harbour), mid-latitude (Sydney) and high-latitude (Tasmania) ranges of genetically distinct clusters within the east coast distribution of *E. radiata* (Minne *et al.*, in prep.; Coleman *et al.*, 2011*b*). Spores were released and gametophytes grown in culture as in Veenhof *et al.* (2022*b*). Cultures were then refreshed monthly with 1 µm filtered, UV-sterilized seawater (FSW) containing quarter strength F growing media (AlgaBoost™ 2000×, AusAqua Pty Ltd, Wallaroo, SA, Australia) under an irradiation of 20 µmol m^–2^ s^–1^ at a 12:12-h cycle and 20, 16 and 12 °C for Coffs Harbour, Sydney and Tasmania, respectively. Cultures were maintained in climatic chambers fitted with LED lamps. These temperatures were based on the approximate average local *in situ* winter temperatures for each population at the time of collection (Wijffels *et al.*, 2018). Coffs Harbour cultures were ~1 year old, Sydney cultures ~2 months old and Tasmania cultures 2 years old before use in this experiment. Before use in this experiment, for each population one culture flask was gently scraped clean with a sterile scraper and blended up to single fragment filaments (~185 ± 15 µm, mean ± s.e.) and diluted at a 1:12 ratio for the final gametophyte stock solution (~27 ± 5 gametophytes mL^–1^). Gametophytes from each population were kept separately and used in independent experiments as below.

### Experimental design

To create a continuous and stable thermal gradient to measure gametophyte responses, we used a purpose-built aluminium thermal gradient block. This aluminium block had 50 wells (41 mm Ø, 25 mm deep) in a 10 × 5 grid. At the far ends of the block were bores through which recirculating hot and cold water flowed continuously. This water was supplied by baths that were cooled using a cooler/heater unit (Teco TK 500) and heated by aquarium heaters (Aqualogic DC series). This system established a stabile thermal gradient resulting in ten temperature treatments along the length of the block that were replicated five times along the breadth of the block. Fifty glass beakers (38 mm Ø) were placed in the temperature block. Each beaker contained 40 mL diluted gametophyte stock solution and two round coverslips (12 mm Ø) as a surface for gametophyte settlement. The opening of each beaker was covered with parafilm to prevent evaporation while allowing sufficient light to penetrate the bottom of the glass beakers (27 µmol m^–2^ s^–1^). Light was supplied via TL fluorescent lamps wrapped in shade cloth, and set at ~27 µmol m^–2^ s^–1^ as measured in the wells covered with parafilm, which is within the light optimum for *E. radiata* gametophytes (Novaczek, 1984*a*). For each population, a different starting temperature was chosen based on the cultures holding temperatures, which were related to average *in situ* winter temperatures at the time of collection to minimize thermal shock (Supplementary Data Fig. S1). The cultures were left for 24 h before starting thermal treatments to allow settlement and attachment to the coverslips and bottom of the glass beakers. One set of replicates (*n *= 5) was kept outside of the temperature block at the respective holding temperatures of 19, 16 and 12 °C and under the experimental light conditions for each population. Abundance, length and sex ratio of these replicates were used as a baseline to calculate survivorship and RGR and sex ratio relative to the starting values for each population (see below). Temperatures in the water baths were altered at a rate of 1–2.5 °C each day (24-h period) depending on the starting temperature, until the final temperatures ranging between 15 and 30.5 °C were reached across the thermal gradient block (Fig. S1). This acclimation period lasted for 7 d for each experiment. Temperatures were checked daily, and light levels monitored weekly throughout the experiment to ensure stability (Light: 26.6 ± 0.14 µmol m^–2^ s^–1^, Temperature: see Fig. S1). In addition, a wireless high-resolution temperature logger was used to measure the lowest (15 °C) and highest (30.5 °C) temperature treatments every 10 min for 4 d, to ensure stability in the temperature block (see Fig. S2). For each population, gametophyte cultures were exposed to experimental temperatures for 14 d.

### Sampling procedure and measurements

At the start of the experiment (baseline measurement) and after 14 d, the response of gametophytes to the thermal gradient was recorded. Cultures were sampled by taking a coverslip, placing it under a glass microscope slide and taking three photos randomly using a MIchrome 20 Color Microscope camera mounted on a stereo microscope (Olympus BX53) at 100× magnification. For each photo (three for each replicate), gametophyte length [averaged over ten individuals per field of view (FOV, 3.2 mm^2^), µm] was measured and the number of individuals (both male and female), as well as number of males, females and juvenile sporophytes per FOV (three for each replicate) was counted. As starting densities and size of gametophytes in each experiment varied slightly, we calculated change relative to the starting density and size, to allow for comparison among experiments and have ecological relevance for spatial modelling. The metrics used were gametophyte relative survival, RGR and male/female ratio.

Survivorship was calculated as number of viable gametophytes (defined as containing at least one pigmented cell) compared to initial number of gametophytes established directly after settlement. RGR was calculated using the following formula (Alsuwaiyan *et al.*, 2021):


$$\begin{array}{l} RGR\left(\;\%\;d^{\text{\textasciitilde{}}\text{-}1}\right)=\left[\left(ln\;L_{2}--\;ln\;L_{1}\right)/\;t_{2}--\;t_{1}\right]\;*100 \end{array}$$

where *L*_1_ and *L*_2_ represent gametophyte length (µm) directly after settlement (*L*_1_) and at the end of the experiment (*L*_2_), and *t* represents the number of days at the start (*t*_1_) and end (*t*_2_) of the experiment. Branch length was measured using ImageJ 1.53e software for ten haphazardly selected individuals.

Due to the destructive nature of sampling, baseline measurements (*t*_1_) were independent of final (*t*_2_) samples. Therefore, RGR values were capped at 0, and survival values were capped at 100.

The male:female ratio was calculated from the total numbers of male and female gametophytes surveyed from three FOV from each replicate coverslip.

### Gametophyte model development

We applied a generalized linear modelling (GLM) framework to assess for the effects of the fixed factor treatment temperature on the survival, RGR and sex ratio of gametophytes of each population. Treatment temperatures as used in the GLM analysis were based on the measured averages (Supplementary Data Fig. S1) but were rounded up at 0.5 °C intervals. GLMs used a logistic link function to model the survival and sex ratio (i.e. binomially distributed response variables), and an identity link function for RGR (i.e. Gaussian distributed response variable). Under-dispersion was detected in the binomial GLMs, so a corrected standard error using a quasi-GLM model was implemented (Zuur *et al.*, 2009). Residual plots were assessed visually to confirm the GLMs satisfied assumptions of homogeneity of variance. Significant effects of temperature were followed by pairwise comparison between sites for every temperature point using estimated marginal means and at a significance level of alpha = 0.05.

For each population, generalized additive models (GAMs) were used to assess the effect of temperature as a continuous variable on gametophyte survival and RGR using the same link functions and distributions as described above. Smoothing functions were removed if the effective degrees of freedom were approximately equal to 1, which is indicative of approximately linear responses (Zuur *et al.*, 2009). All analyses were undertaken in R (R Core Team, 2022) using the *mgcv* package (Wood *et al.*, 2016) to fit all GAMs and GLMs. Pairwise comparisons were done using the package *emmeans* (Length, 2022), and plots were generated with the packages *ggplot2* (Wickham, 2016) and *visreg* (Breheny and Burchett, 2017).

### Spatial analyses

Historical spatial predictions and future spatial projections were made for gametophyte survival and RGR using the population-specific GAMs and GLMs that related these response variables to temperature. Sex ratio was not included in spatial analyses as no significant effect of temperature on sex ratio was identified for the Sydney population. Spatial analyses were restricted to depths shallower than 50 m, using depth data from the General Bathymetric Chart of the Oceans (GEBCO_2020) dataset. Historical predictions and future projections were created for three distinct regions using unique models fitted to data obtained from the genetically distinct *E. radiata* populations that occur within each region. Specifically, models fitted to thermal response data from the low-latitude Coffs Harbour population were projected to 28°S–31°S, models fitted to data from the mid-latitude Sydney population were projected to 34.5°S–31°S and models fitted to data from the high-latitude Tasmania population were projected to 41°S–44°S.

Historical predictions were made for each season and population and were averaged over a 28-year period encompassing 1994–2022. Temperature data used in the historical predictions of gametophyte survival and RGR were obtained from the reprocessed (L4) Operational SST and Ice Analysis (OSTIA) system (Good *et al.*, 2020), downloaded from the Copernicus Marine Environment Monitoring Service (https://marine.copernicus.eu; product #010_011) and had a 0.05° spatial resolution. Daily measures of sea surface temperature (SST) were seasonally aggregated and averaged over a 28-year period (encompassing 1994–2022).

Future projections for each season and population were centred on a near-future 2050 (2040–2059 period). Temperature data used in future projections of gametophyte survival and RGR were obtained from six global climate models (GCMs) forced under RCP4.5 and 8.5 emissions scenarios from the Coupled Model Intercomparison Project (CMIP5; Table S1). Given existing variability among GCMs (Drenkard *et al.*, 2021), we used a multi-model ensemble of SST data consisting of the average of all six models to provide a robust estimate of future ocean conditions off eastern Australia. To match the spatial resolution of our historical predictions (0.05°), we used the delta ‘change-factor’ method (e.g. Morley *et al.*, 2018; Navarro-Racines *et al.*, 2020; von Hammerstein *et al.*, 2022) to downscale SST from ~1° to a common 0.05° spatial resolution. Delta downscaling has the advantage of providing high-resolution temperature data over decadal time periods while being relatively simple in application (Navarro-Racines *et al.*, 2020; Drenkard *et al.*, 2021). In addition, this method reduces model bias by use of high-resolution observed data to generate future temperature data, thus including empirical data on small-scale variations into the final model (Pourmokhtarian *et al.*, 2016).

The delta downscaling process involved (1) remapping the curvilinear source GCM temperature data to a global 1° rectilinear grid using the second-order conservative algorithm (remapcon2) in Climate Data Operators (Schulzweida, 2021); (2) infilling missing data adjacent to the continental coast for datasets describing zonal (U) and meridional (V) flows using thin plated splines interpolation in R (R Core Team, 2022); (3) calculating the difference (i.e. delta value) between seasonally aggregated temperature data for the period 2040–2059 and a modelled historical baseline period encompassing 1993–2012 for each GCM and RCP scenario; (4) disaggregating delta value matrices from their native model resolution (~1°) to the finer resolution of observed ocean data (i.e. 0.05°) using bilinear interpolation; and (5) adding delta values to the observed seasonal temperature data encompassing the period 1993–2012. This method produced future ocean temperature data, seasonally aggregated and downscaled to a common 0.05° resolution for the period 2040–2059 which was used to generate future projections of gametophyte survival and RGR.

Finally, the historical predictions were used to calculate the projected change between historical and future periods for gametophyte survival and RGR for the three genetically distinct populations. Future projections under RCP4.5 are presented in the Supplementary Data, while projections under RCP8.5 are presented in the main text as this scenario most closely aligns with the current trajectory of climate change (Schwalm *et al.*, 2020). Spatial analyses were undertaken using the *raster* package (Hijmans and Etten, 2012) and maps were generated using the *tmap* package (Tennekes, 2018) in R (R CoreTeam, 2022).

## RESULTS

### Effects of temperature on gametophyte survival, RGR and sex ratio on the populations

**Fig. 1.**
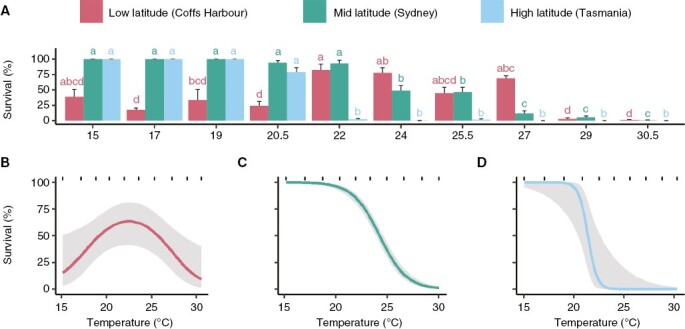
Survival of *E. radiata* gametophytes of genetically separate populations. (A) Mean percentage survival of each population over ten temperatures ranging from 15 to 30.5 °C. Error bars represent standard error (*n* = 5). Letters denote significant differences (one-way ANOVA for each population, post-hoc pairwise test). (B) Effect of temperature on the fitted values of the optimal model for the survival of Coffs Harbour (low-latitude) gametophytes, (C) Sydney (mid-latitude) gametophytes and (D) Tasmania (high-latitude) gametophytes. For all three model plots (B–D), grey areas denote the 95 % confidence interval and rugs (short stripes) represent the observed data.

Survival varied among temperatures for each population in *E. radiata* gametophytes (Table 1; Fig. 1A). Pairwise comparison showed that the low-latitude gametophytes had a lower overall survival rate between 15 and 20.5 °C (13–37 %) compared to the survival between 22 and 27 °C (45–82 % *P* < 0.05, Fig. 1A). In contrast, mid- and high-latitude gametophytes had highest survival from 15 to 20.5 °C (79–100 %) compared to higher temperatures (*P* < 0.05, Fig. 1A). Survival was significantly lowered for low-latitude gametophytes from 29 °C (<10 %, *P* < 0.05, Fig. 1A). Mid-latitude gametophytes had a significantly lower survival from 24 °C (49 %), which was significantly lower again from 27 °C (<10 %, *P* < 0.05, Fig. 1A). Survival was significantly lower at temperatures above 22 °C for high-latitude gametophytes (<2.5 %, *P* < 0.05, Fig. 1A). At 29 and 30.5 °C, there was no survival for high-latitude gametophytes, while 1–6 % of gametophytes from low and mid-latitudes survived in the two highest temperature treatments (Fig. 1A).

**Table 1.** Results of one-way ANOVAs on the survival, RGR and sex ratio of *E. radiata* gametophytes using fixed factor Temperature (levels: 15, 17, 19, 20.5, 22, 24, 25.5, 27, 29, 30.5 °C) for each population (Coffs Harbour, Sydney, Tasmania). Bold printed p-values signify significance at the α ≤ 0.05 level.

ANOVAs for each population of the effect of treatment temperature					
Population	Response variable	Factor	d.f.	*F*	*P*-value
Coffs Harbour	Survival	Temperature	9	11.25	**<0.001**
		Residuals	40		
	RGR	Temperature	9	10.97	**<0.001**
		Residuals	39		
	Sex ratio	Temperature	9	22.66	**<0.001**
		Residuals	37		
Sydney	Survival	Temperature	9	67.97	**<0.001**
		Residuals	40		
	RGR	Temperature	9	37.55	**<0.001**
		Residuals	38		
	Sex ratio	Temperature	9	0.15	0.997
		Residuals	37		
Tasmania	Survival	Temperature	9	292.69	**<0.001**
		Residuals	40		
	RGR	Temperature	6	7.57	**<0.001**
		Residuals	20		
	Sex ratio	Temperature	5	31.30	**<0.001**
		Residuals	18		

Differential survival among gametophyte populations resulted in unique survival curves for each population (Fig. 1B–D). For the low-latitude population, a GAM with temperature as a significant (*P* = 0.03) explanatory variable resulted in a bell-shaped curve with maximum survival around 23 °C, and lowest survival at either extreme of 15 and 30 °C (Fig. 1B). In contrast, smoothers were dropped in favour of a linear model with a temperature as a significant predictor for both mid- (*P* < 0.001, coefficient estimate: -0.7687, Fig. 1C) and high-latitude populations (*P* = 0.01, coefficient estimate: −2.19, Fig. 1D). Mid-latitude gametophytes had a 100 % survival rate up to 20 °C, where survival decreased gradually with temperature until 30 °C (Fig. 1C). High-latitude gametophytes survived up to 20 °C, and then survival decreased steeply to 0 % at 23 °C (Fig. 1D).

**Fig. 2.**
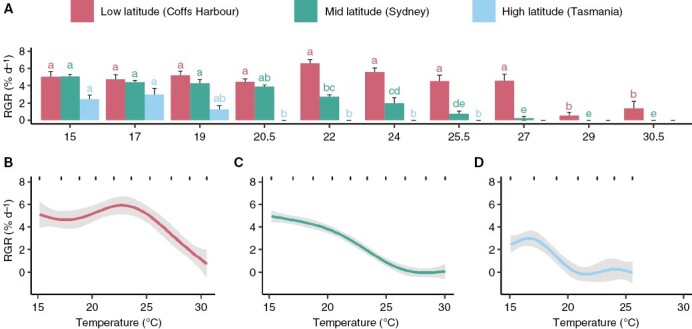
Relative growth rate (RGR, % d^−1^) of *E. radiata* gametophytes of genetically separate populations. (A) Mean RGR of each population over ten temperatures ranging from 15 to 30.5 °C. Error bars represent standard error (*n* = 5, but from 22 °C onward the Tasmanian gametophytes are associated with fewer replicates due to death of gametophytes, as were the Coffs Harbour and Sydney gametophytes at 30.5 °C). Letters denote significant differences (one-way ANOVA for each population, post-hoc pairwise test). (B) Effect of temperature on the fitted values of the optimal model for the survival of Coffs Harbour (low-latitude) gametophytes, (C) Sydney (mid-latitude) gametophytes and (D) Tasmania (high-latitude) gametophytes. For all three model plots (B–D), grey areas denote the 95 % confidence interval and rugs (short stripes) represent the observed data.

RGR of kelp gametophytes was found to vary with temperature for each population (Table 1; Fig. 2A). Low-latitude gametophytes maintained a relatively high RGR (4.5–5.5 % d^−1^) up to 27 °C. Pairwise comparison showed that low-latitude gametophytes had a significantly lower RGR between 29 and 30.5 °C compared to lower temperatures (*P* < 0.05, Fig. 2A). Despite this, low-latitude populations maintained positive RGR (0.5–1.5 % d^−1^) until 30.5 °C (Fig. 2A). Mid-latitude gametophytes had a lower RGR from 22 °C (2.7 % d^−1^) and RGR was lowest from 27 °C onward (<1 % d^−1^, *P* < 0.05, Fig. 2A). High-latitude populations had a significantly lower RGR from 20.5 °C onward (<1 % d^−1^, *P* < 0.05, Fig. 2A). High-latitude gametophytes did not survive past 25.5 °C and therefore no RGR is shown at values higher than this.

Differential RGRs among gametophyte populations resulted in unique RGR curves for each population (Fig. 2B–D). For low-latitude gametophytes, a GAM with temperature as a significant (*P* < 0.001) explanatory variable revealed gametophytes had an RGR of ~5 % d^−1^ between 15 and 22 °C, peaking with an RGR of 6 % d^−1^ at 23 °C and then gradually declining to ~1 % d^−1^ at 30 °C (Fig. 2B). For mid-latitude gametophytes, a GAM with temperature as a significant (*P* < 0.001) explanatory variable showed the highest RGR of 5 % d^−1^ occurred at 15 °C, gradually declining to an RGR of 0 % d^−1^ at 30 °C (Fig. 2C). For high-latitude gametophytes, a GAM with temperature as a significant (*P* < 0.001) explanatory variable resulted in an RGR of 4 % d^−1^ between 15 and 17 °C and declining to 0 % d^−1^ between 20 and 25 °C, after which no gametophytes survived (Fig. 2D).

**Fig. 3.**
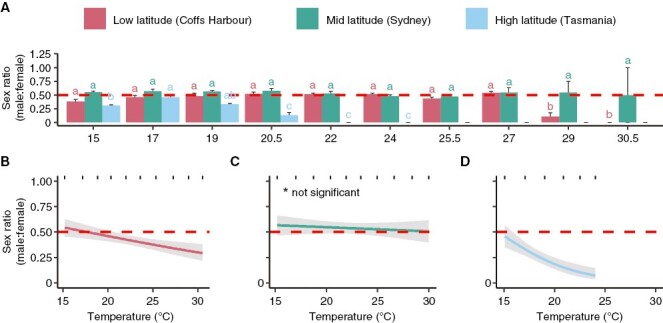
Sex ratio (male:female) of *E. radiata* gametophytes of genetically separate populations. (A) Mean sex ratio of each population over ten temperatures ranging from 15 to 30.5°. Error bars represent standard error (*n* = 5, but from 22 °C onward the Tasmanian gametophytes are associated with fewer replicates due to death of gametophytes, as were the Coffs Harbour and Sydney gametophytes at 30.5 °C). Letters denote significant differences (one-way ANOVA for each population, post-hoc pairwise test). (B) Effect of temperature on the fitted values of the optimal model for the sex ratio of Coffs Harbour (low-latitude) gametophytes, (C) Sydney (mid-latitude) gametophytes and( D) Tasmania (high-latitude) gametophytes. The red dotted line denotes a 0.5 sex ratio, where there is an equal number of males and females. For all three model plots (B–D), grey areas denote the 95 % confidence interval and rugs (short stripes) represent the observed data.

Sex ratios of kelp gametophytes were influenced by temperature in each population (Table 1; Fig. 3A). Pairwise comparison showed a significant decrease in male gametophytes in the low-latitude population at 29 and 30.5 °C compared to lower temperatures (*P* < 0.05, Fig. 3A). Mid-latitude gametophytes stayed at an approximate equal sex ratio (Fig. 3A). High-latitude gametophytes had a skew towards females at 15 °C (*P* < 0.05) and 19 °C (*P* < 0.05, Fig. 3A). High-latitude gametophyte sex ratio was significantly lower compared to to higher temperatures of 20.5–24 °C (*P* < 0.05, Fig. 3A), resulting in significantly fewer males occurring within high-latitude gametophytes.

Differential sex ratios resulted in separate sex ratio curves for each population (Fig. 3B–D). For all populations, smoothers were dropped in favour of linear models with temperature as a significant linear predictor for low- (*P* = 0.003, coefficient estimate: −0.069, Fig. 3B) and high-latitude gametophyte sex ratio (*P* < 0.001, coefficient estimate: −0.264, Fig. 3D), while temperature was not a significant predictor of sex ratio for mid-latitude gametophytes (Fig. 3C). Both low- and high-latitude gametophytes have an equal (0.5) sex ratio at relatively low temperatures, and the sex ratio (male:female) decreased with increasing temperatures (Fig. 3B, D). The sex ratio of 0.5 in mid-latitude gametophytes is unaffected by temperature (Fig. 3C).

### Projected survival and RGR under future ocean warming

**Fig. 4.**
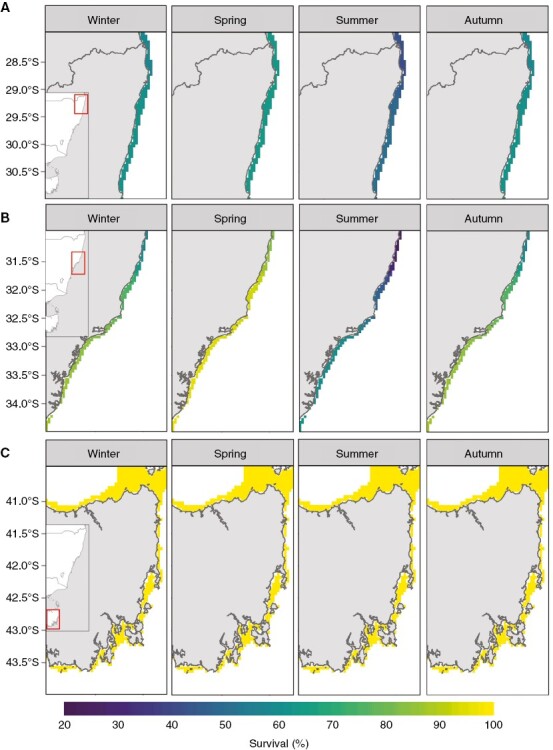
Spatial projections of the change in (A) survival (left panels) and (B) RGR (% d^−1^, right panels) between the historical baseline (encompassing monthly averaged aggregated to annual averages from 1994 to 2022) and future climate scenario RCP8.5 centred around 2050-future (2040–2059 period). The three populations are low latitude (Coffs Harbour, 28°S–31°S), mid-latitude (Sydney, 31°S–34.5°S) and high latitude (Tasmania, 41.5°S–44°S).

Projections of gametophyte survival and RGR varied seasonally and latitudinally under ocean warming. Relative to present-day conditions, survival of gametophytes is projected to decline by 20–30 % in the mid-latitude population by 2050 under RCP8.5 (Fig. 4A). Relatively lower declines in survival (0–10 %) are projected for the low-latitude populations, and no change in survival for the high-latitude populations by 2050 under this climate change scenario (Fig. 4A).

**Fig. 5.**
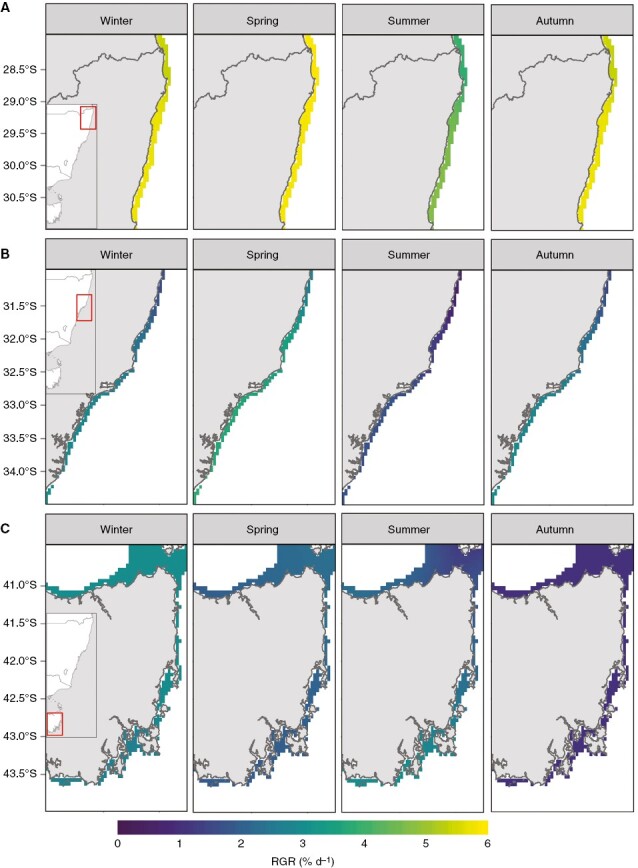
Survival of *E. radiata* gametophytes among three separate populations for a 2050-centred future period (2040–2059) under climate scenario RCP8.5. The three populations are (A) low latitude (Coffs Harbour, 28°S–31°S), (B) mid-latitude (Sydney, 31°S–34.5°S) and (C) high latitude (Tasmania, 41.5°S–44°S). Monthly projections have been seasonally aggregated (Winter = June to August, Spring = September to November, Summer = December to February, Autumn = March to May).

**Fig. 6.**
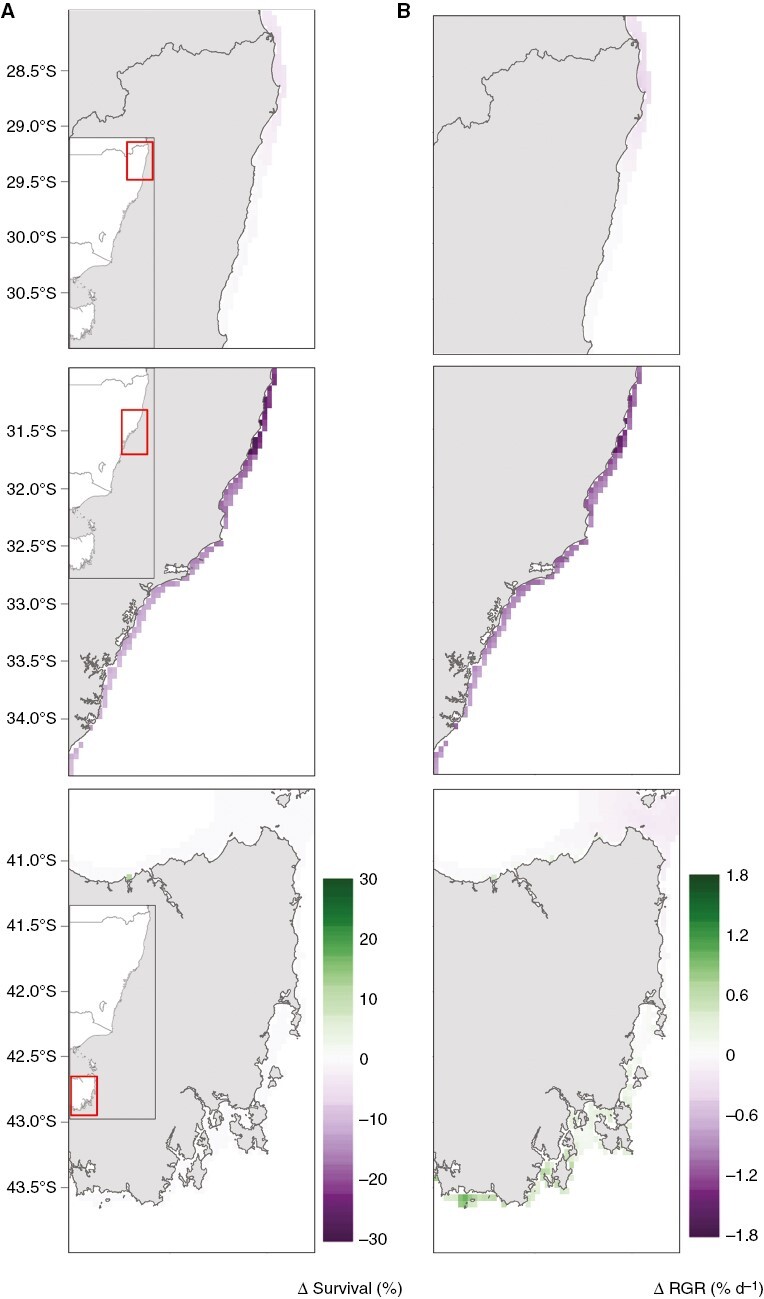
Relative growth rate (RGR, % d^−1^) of *E. radiata* gametophytes among three separate populations for a 2050-centred future period (2040–2059) under climate scenario RCP8.5. The three populations are (A) low latitude (Coffs Harbour, 28°S–31°S), (B) mid-latitude (Sydney, 31°S-34.5°S) and (C) high latitude (Tasmania, 41.5°S–44°S). Monthly projections have been seasonally aggregated (Winter = June to August, Spring = September to November, Summer = December to February, Autumn = March to May).

Generally, projections under RCP8.5 and RCP4.5 were consistent, but the magnitude and direction of changes were intensified under RCP8.5 (Figs. 5 and 6; Supplementary Data Figs S3 and S4 present projected survival and RGR under RCP4.5, respectively). Overall survival rates of the lower latitude population (28°S–31°S) were projected to range between 70 and 40 % in 2050 under RCP8.5 (Fig. 5A), which was lower than for the mid- and high-latitude populations (Fig. 5B, C). Lowest survival rates in the low-latitude population were projected in summer (~40 %), and this was intensified under RCP8.5 (Fig. 5A; Fig. S3). The highest survival rates in the low-latitude population were projected in spring under RCP8.5, with up to 70 % survival (Fig. 5A). The mid-latitude populations (31°S–34.5°S) had a broader projected range of survival rates under RCP8.5, between 20 and 100 % (Fig. 5B), as well as more pronounced latitudinal differences in projected survival compared to the other two populations (Fig. 5). Stark seasonal differences in projected survival became apparent in the mid-lattitude population under RCP8.5, where summer survival was projected to be as low as 20 %, while survival was projected between 80 and 100 % in spring under RCP8.5 (Fig. 5B). The high-latitude populations (41.5°S–44°S) had a projected survival rate of 100 % across seasons and latitude under RCP8.5 (Fig. 5C).

RGR was projected to be higher within the lower latitude population (28°S–31°S; 4–6 % d^−1^) over all seasons, compared to the other populations by 2050 under RCP8.5 (Fig. 6). Relative to present day levels, RGR was projected to decline between 1.8 and 1.2 % d^−1^ in the mid-latitude population by 2050 under RCP8.5 (Fig. 4B). Relatively lower declines in RGR (0–0.5 % d^−1^) are projected for the low-latitude populations (Fig. 4B). An increase in RGR of up to 1.2 % d^−1^ was projected for the high-latitude population under RCP8.5 in the southern regions, while a small decrease in RGR (>0.5 % d^−1^) was projected in the northern regions of that population under the same ocean warming scenario (Fig. 4B).

The low-latitude population had the lowest projected RGR in summer (4 % d^−1^), which was slightly more pronounced under RCP8.5 (Fig. 6A; Supplementary Data Fig. S4). The mid-latitude population (31°S–34.5°S) also had lower projected RGRs in summer under RCP8.5 with RGR of near 0 % d^−1^ in the northmost regions (Fig. 6B). RGRs were projected to be highest in spring for the mid-latitude population under RCP8.5, between 4 and 5 % d^−1^ (Fig. 6B). The high-latitude population (41.5°S–44°S) had the lowest projected RGRs under RCP8.5 compared to the other two populations (Fig. 6), ranging between 1 and 3 % d^−1^ (Fig. 6C). The starkest contrast between RCPs was projected in this population for autumn, where a relatively high RGR was projected under RCP4.5 (3 % d^−1^, Fig. S3), but under RCP8.5, RGR was projected to be nearly 0 % d^−1^ (Fig. 6C). Winter had high projected RGRs under RCP8.5 for the high-latitude population (4 % d^−1^), while a latitudinal difference in projected RGR became apparent in summer, with lower projected RGRs in the northern ranges of the population (Fig. 6C).

## DISCUSSION

Kelp forests are projected to decline as ocean warming increases (Wernberg *et al.*, 2019*b*). However, these projections fail to consider the microscopic gametophyte stage of kelps, which often has a higher thermal tolerance than adult plants (tom Dieck, 1993; Veenhof *et al.*, 2022*a*), or spatial variation in kelp thermal tolerance. Here, for the first time, we used laboratory-derived thermal performance data for *E. radiata* gametophytes to model and project changes in the survival and RGR of *E. radiata* gametophytes along eastern Australia.

Many kelp gametophyte populations show variability in their thermal response, which can suggest adaptation to local temperatures (Mabin *et al.*, 2019; Muth *et al.*, 2019; Liesner *et al.*, 2020; Alsuwaiyan *et al.*, 2021). This ability to adapt to local temperatures ensures kelps can thrive in a broad range of temperature conditions (Müller *et al.*, 2008; King *et al.*, 2018). We found evidence for thermal plasticity among populations of *E. radiata* gametophytes, which was related to local temperatures. Survival was higher for the low-latitude gametophytes in the warmer temperature treatments (22.5–27 °C), reflecting the temperatures this population experiences in the field (17–27 °C; Wijffels *et al.*, 2018). Similarly, the upper survival and RGR limit for mid- and high-latitude gametophytes was 25.5 and 20.5 °C, respectively, which was congruent with local temperature ranges of 15–23 °C for the mid-latitudes and 11–18 °C for high latitudes (Wijffels *et al.*, 2018). Thermal plasticity in accordance with local temperatures among geographically distinct populations of gametophytes has also been found on the west coast of Australia in *E. radiata* (Mohring *et al.*, 2014), and among populations in *Macrocystis pyrifera* (Hollarsmith *et al.*, 2020) and *Laminaria digitata* (Liesner *et al.*, 2020, Martins *et al.*, 2020). As we used different holding temperatures (*in situ* winter temperatures) for each gametophyte population to have ecologically realistic data for modelling purposes, we cannot untangle the influence of recent environmental history from an underlying genetic basis in the thermal tolerance expressed in this experiment. However, genotype × environment interactions have been found in *E. radiata* (Mabin *et al.*, 2019, Alsuwaiyan *et al.*, 2021) and hence our results are likely due to both phenotypic plasticity and underlying genetic variation related to thermal traits.

*Ecklonia radiata* gametophytes from the low-latitude population had the highest thermal tolerance recorded for kelp gametophytes in any study (maintaining positive RGRs up to 30.5 °C), akin to warm-water-adapted kelp species (monthly means above 15 °C in summer; Veenhof *et al.*, 2022*a*) *Eisenia bicyclis* and *Undaria pinnatifida* (tom Dieck, 1993). Notably, each population had a thermal tolerance range that exceeds their maximum local temperature by ~2–3 °C, meaning none of the populations are nearing their thermal maxima within their current distributions. Thermally tolerant gametophytes are common in warm-adapted kelp species, such as *Ecklonia radicosa* (Komazawa *et al.*, 2015), where maximum survival temperatures are higher than current *in situ* temperatures (tom Dieck, 1993, Veenhof *et al.*, 2022*a*). This thermal tolerance may play an important role in resilience to future warming, though some southern hemisphere gametophytes are at their current thermal limit (Paine *et al.*, 2021). Despite the thermal resilience of gametophyte survival and growth, the transition from gametophyte to sporophyte stages can be more sensitive to temperature (Martins *et al.*, 2020; Veenhof *et al.*, 2022*a*).

The proportion of males to females in gametophyte populations can vary depending on environmental conditions (Bolton and Lüning, 1982; Oppliger *et al.*, 2011; Martins *et al.*, 2020; Veenhof *et al.*, 2022*a*). A skewed sex ratio is often caused by post-germination mortality of either males or females in response to environmental stress such as light and temperature (Xu *et al.*, 2015; Martins *et al.*, 2020). *Ecklonia radiata* gametophytes in this study skewed sex ratios to favour more females when temperatures approached the population’s thermal maximum, but only for the two range-edge populations. Increased survival of females under increased temperature at range edges has also been observed in *Lessonia nigrescens* (Oppliger *et al.*, 2011) and *Macrocystis pyrifera* (Rodriguez *et al.*, 2019). This contrasts with other, non-range-edge populations, where survival of males is higher under temperature stress (e.g. Bolton and Lüning, 1982; Komazawa *et al.*, 2015; Martins *et al.*, 2020). An increased number of females at lower latitudes may help increase fecundity, as the number and size of eggs per female can decrease in low-latitude gametophyte populations (Camus *et al.*, 2021) as well as with increased temperature stress (Müller *et al.*, 2008; Martins *et al.*, 2017; Hollarsmith *et al.*, 2020). An increased number of females may also result in parthenogenesis, asexual reproduction through female gametophytes, which can occur in range edges in response to marginal conditions (Oppliger *et al.*, 2014).

Incorporating thermal tolerances of different populations can considerably improve projections of future survival for kelps (King *et al.*, 2018). While the projected survival for the low-latitude gametophyte population ranged between 30 and 70 %, their projected RGRs were the highest among populations. Conversely, high-latitude populations had the lowest projected RGRs, down to 0 in autumn. This may indicate separate trade-offs between survival and growth for lower and higher latitude populations. Low survival but high RGRs at higher temperatures cause fewer, but larger gametophytes in the low-latitude populations, and high survival but low RGRs at higher temperatures cause a greater number of smaller gametophytes for the high-latitude population. A similar trade-off to that of the low-latitude populations under increased temperatures (lower survival but increased growth) has been observed for *E. radiata* gametophytes across Western Australia (Mohring *et al.*, 2013). Larger female gametophytes produce more eggs per female (Muñoz *et al.*, 2004; Camus *et al.*, 2021), and this may compensate for reduced fertility resulting from higher mortality and lowered fecundity in the low-latitude population (Camus *et al.*, 2021). The timing of spore supply may also compensate for the lowered projected survival in summer for mid- and low-latitude populations. Spore supply peaks in autumn in mid-latitude populations (Mabin *et al.*, 2013) and spore supply is available year-round in high-latitude populations (Veenhof *et al.*, 2023). Any gametophyte die-off during hotter summer months can thus be replenished with fresh spores in autumn. Spore supply also peaks in autumn for high-latitude populations (Tatsumi *et al.*, 2021), meaning under an RCP8.5 2050 future RGRs will be lowest when the highest spore release occurs. However, fertility peaks may also shift due to ocean warming and may result in a mismatch between peak spore supply and optimum gametophyte survival and RGR (Martins *et al.*, 2017).

Models using data on adult *E. radiata* sporophytes have projected a range contraction of ~275 km in the area that corresponds with our low- and mid-latitude population by 2100 under RCP8.5 (Davis *et al.*, 2022). Projections based on gametophyte models revealed minimal changes in survival compared to historical predictions. In addition, thermal adaptation of gametophytes may lead to higher survival than the projected survival rates, making even these minimal projections conservative (Mabin *et al.*, 2019; Becheler *et al.*, 2022). The mid-latitude population was identified as the most vulnerable, with a projected 30 % decrease in survival. This is due to a relatively lower thermal tolerance of that population compared to the low-latitude population, while some parts of the mid-latitude population would experience temperature increases similar to the low-latitude population. However, the low-latitude population had a projected 0 % change in survival, and high connectivity among *E. radiata* populations along eastern Australia (Coleman *et al.*, 2011*a*, *b*, 2017) may result in more thermally tolerant gametophytes migrating to higher latitudes, potentially increasing projected survival rates of the mid-latitude population.

While sporophyte populations are projected to decline, gametophyte populations may act as a buffer during periods of high temperature stress such as summers and marine heatwaves and facilitate rapid recovery. Gametophytes can persist in the form of vegetatively growing, mixed-origin seedbanks persisting through temperature extremes of up to 27–30 °C in the lower latitudes (*sensu*Carney *et al.*, 2013). The exact temperature window for transition to sporophytes is not known for the separate populations, though it is likely between 16 and 20 °C in high-latitude populations (from 34°S and higher; Novaczek, 1984*b*; Mabin *et al.*, 2013; Mohring *et al.*, 2014; Alsuwaiyan *et al.*, 2021). Recruitment patterns may shift in *Ecklonia* during the hot summer months when sporophyte loss is more likely to occur, but surviving gametophytes may facilitate recruitment and repopulation in winter and spring. Integrating the exact temperature thresholds of recruitment (transition from gametophyte into sporophyte) across populations into kelp models may more accurately project future distributions of kelp populations (Assis *et al.*, 2017; Paine *et al.*, 2021), which is an important avenue of future research.

## CONCLUSION

Kelp forest persistence is threatened by ocean warming globally. Here we reveal, via population-specific gametophyte models, that *E. radiata* gametophytes have thermal tolerances in accordance with their local temperatures across 13° of latitude along the east coast of Australia. They also have a thermal tolerance of ~2–3 °C above their local thermal maxima, indicating that the gametophyte stage of *E. radiata* is likely to be resilient to near future ocean warming and may provide population resilience as a thermally tolerant seedbank. However, mismatches between peak spore supply and optimum survival may occur under future warming scenarios. It will also be crucial to pinpoint the exact temperatures of stage transitions from gametophyte to sporophyte to fully understand the effects of ocean warming on kelp persistence. Projections of future kelp loss should consider local thermal adaptation, as well as the thermal resilience of the gametophyte stage to provide more accurate outcomes.

## SUPPLEMENTARY DATA

Supplementary data are available online at https://academic.oup.com/aob and consist of the following.

Figure S1. Treatment temperatures during the thermal experiment for populations Coffs Harbour, Sydney and Tasmania. Figure S2. Temperature logger data recording temperature every 10 min for the 15 and 30.5 °C treatment for 4 d. Figure S3. Survival of *E. radiata* gametophytes among three separate populations for a 2050-centred future period under climate scenario RCP4.5. Figure S4. Relative growth rate of *E. radiata* gametophytes among three separate populations for a 2050-centred future period under climate scenario RCP4.5. Table S1. Details of GCMs (CMIP5) downscaled (0.05°) to support projections gametophyte survival and RGR off eastern Australia. The downscaled variable from each model include was sea surface temperature under RCP4.5 and 8.5 emissions scenarios

mcad132_suppl_Supplementary_Data

mcad132_suppl_Supplementary_Figures_S1-S4

mcad132_suppl_Supplementary_Table_S1
