# Supplementary material for: Projecting kelp (Ecklonia radiata) gametophyte thermal adaptation and persistence under climate change
Source: Ann Bot. 2023 Sep 4;133(1):153–68. doi: 10.1093/aob/mcad132 (PMC10921825; doi:10.1093/aob/mcad132)
Supplement: mcad132_suppl_Supplementary_Figures_S1-S4 [file mcad132_suppl_supplementary_figures_s1-s4.docx]

**Supplementary material – Figures S1-S3**


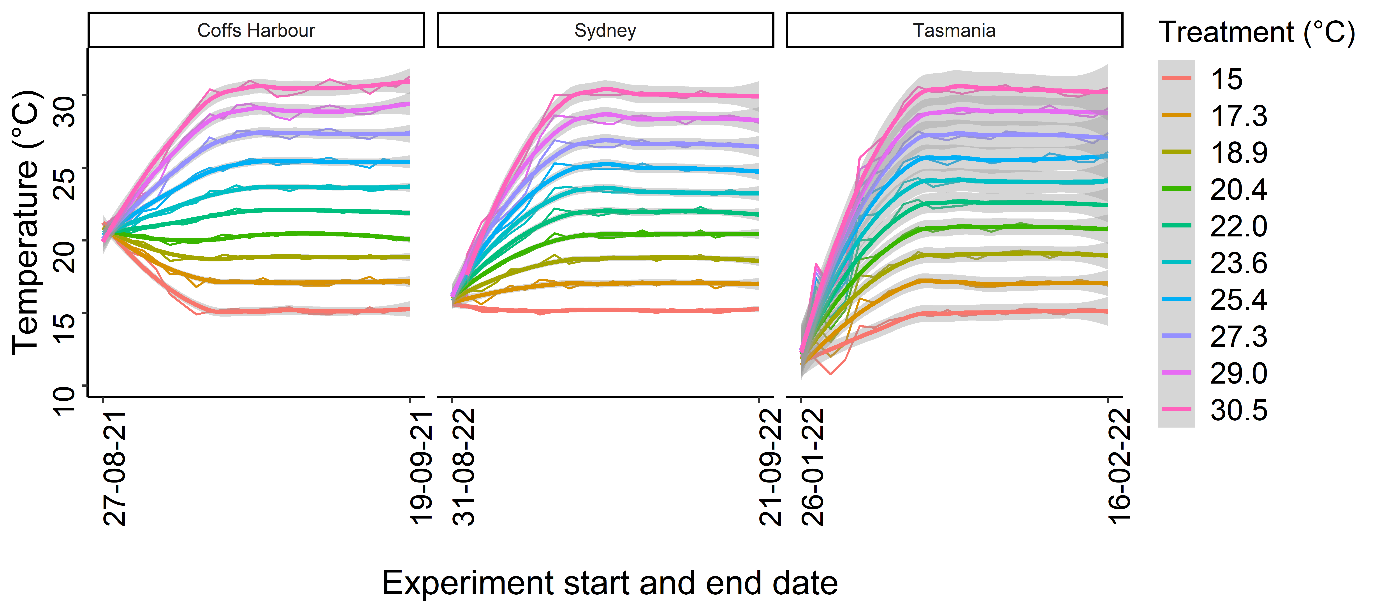


Figure S1. Treatment temperatures during the thermal experiment for populations Coffs Harbour (low latitude), Sydney (mid latitude) and Tasmania (high latitude). Each population was started at temperatures corresponding to *in situ* winter temperatures, 20°C, 16°C and 12°C, respectively. Temperatures were ramped up to experimental temperatures (Treatment) over a 7-day period and maintained for 2 weeks.


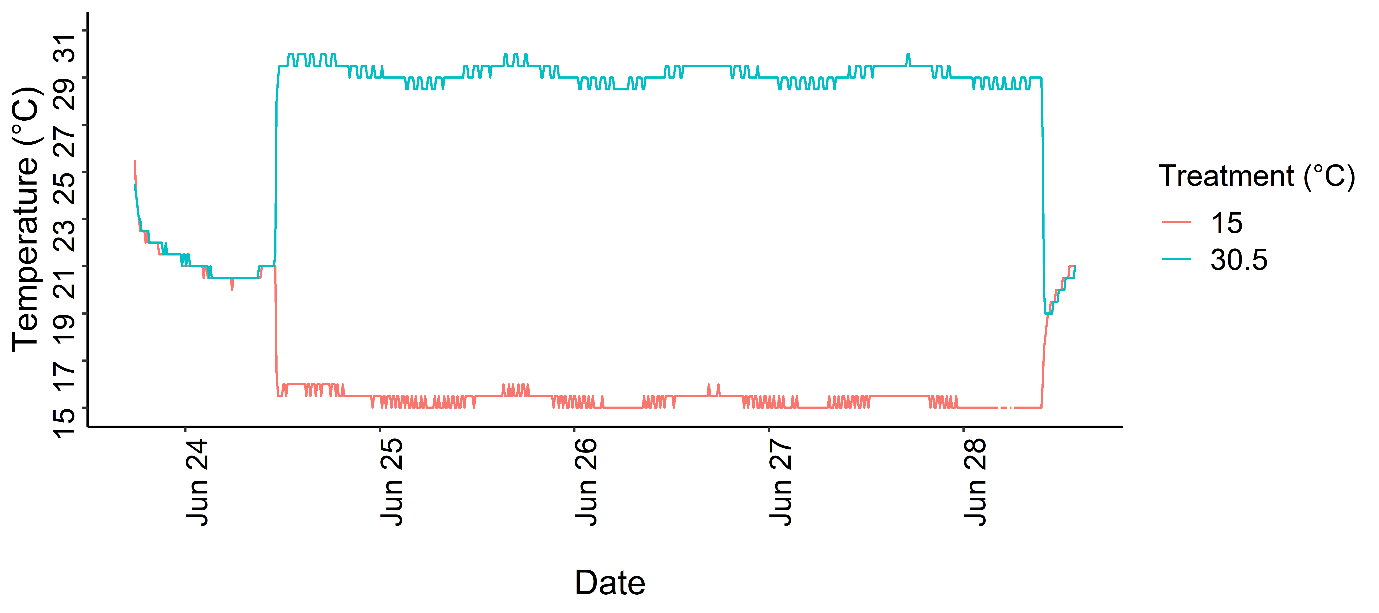
Figure S2. Temperature logger data recording temperature every 10 minutes for the 15°C and 30.5°C treatment for 4 days, showing stable temperatures during day and night when using the aluminium temperature block.


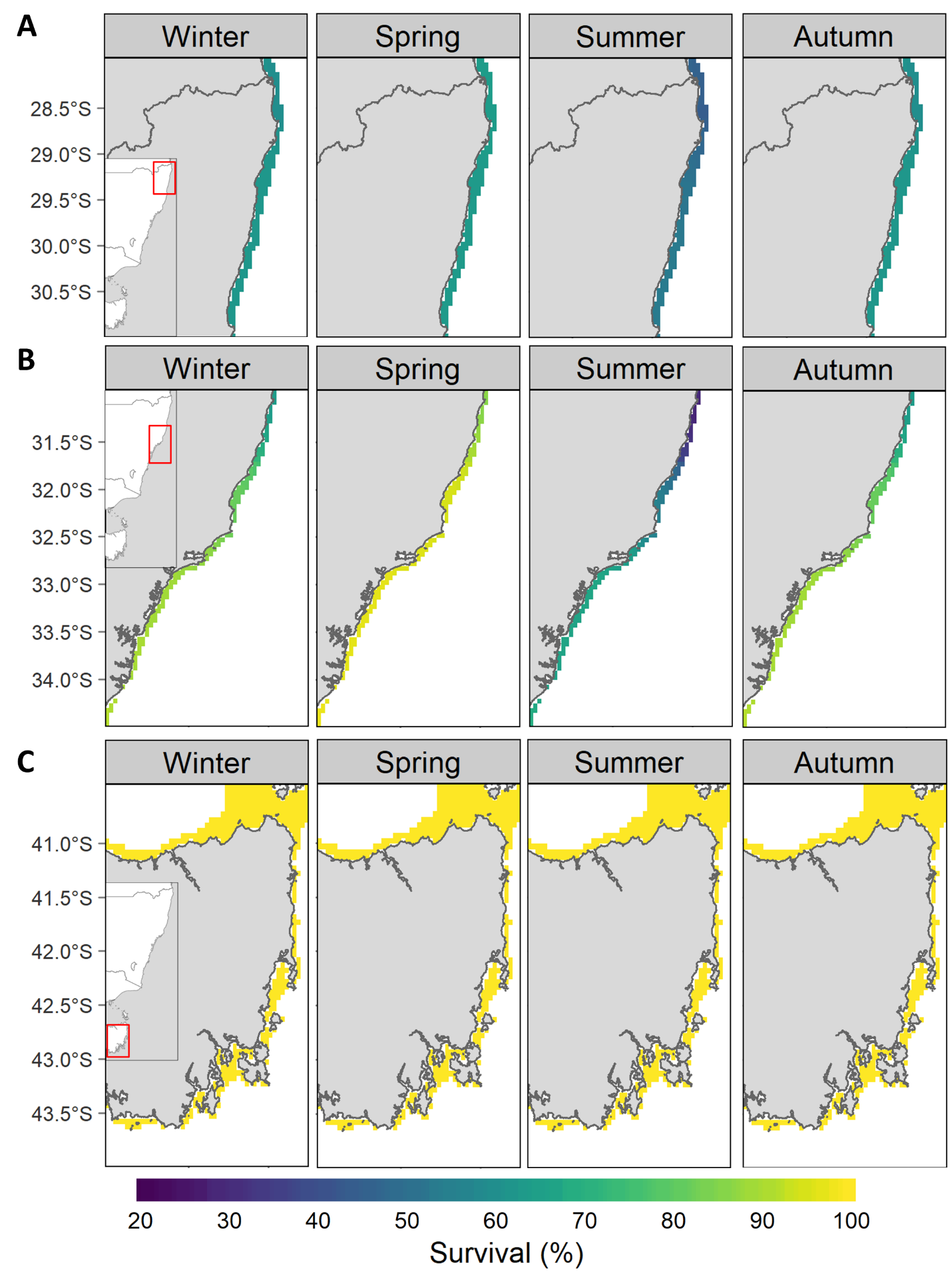


Figure S3. Survival of *E. radiata* gametophytes among three separate populations for a 2050-centered future period (2040-2059) under climate scneario RCP4.5. The three populations are Coffs Harbour (low latitude, 28°S-31°S), Sydney (mid latitude, 31°S-34.5°S) and Tasmania (high latitude, 41.5°S-44°S). Monthly projections have been seasonally aggregated (Winter = June to August, Spring = September to November, Summer = December to February, Autumn = March to May).


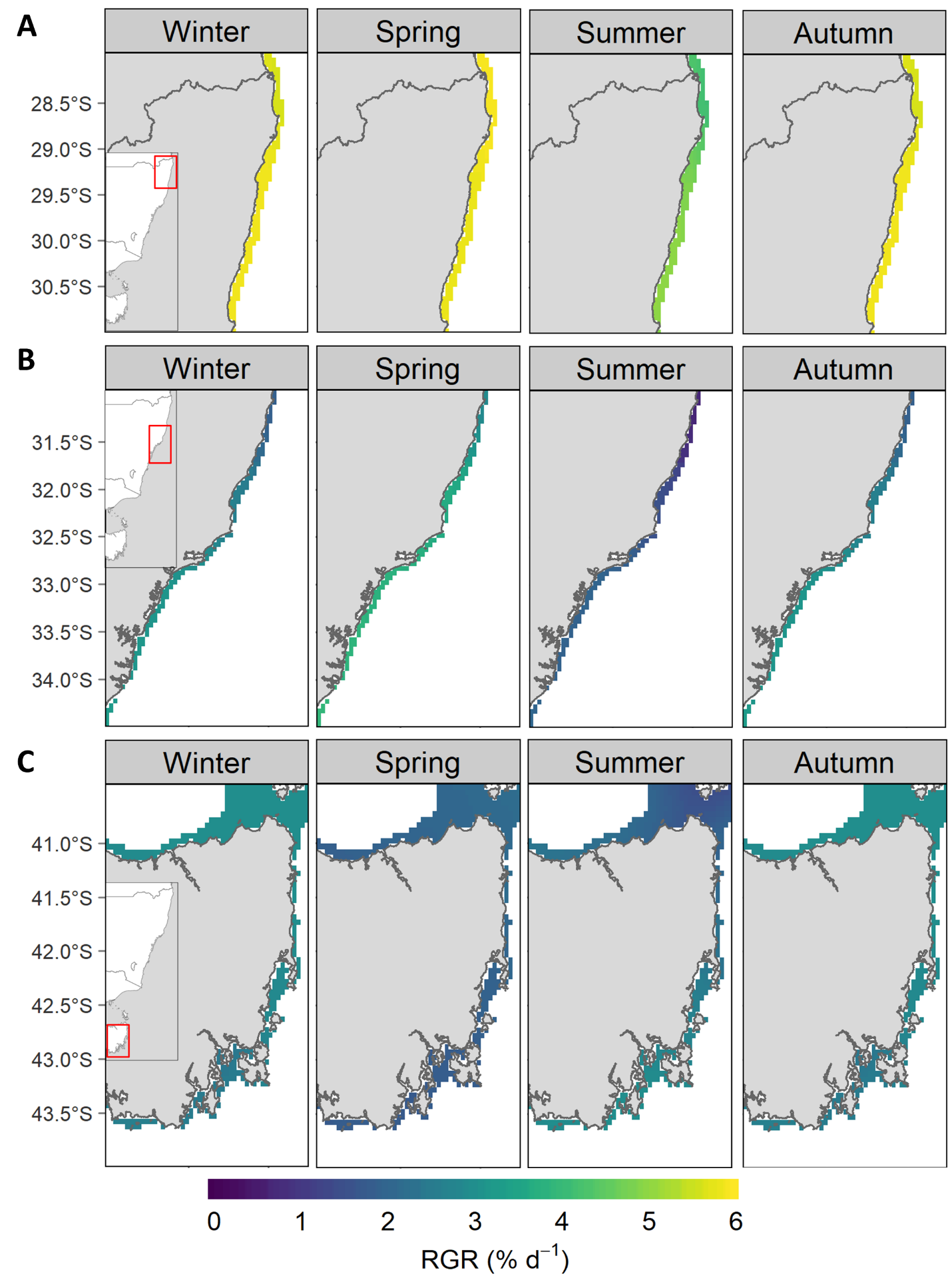
Figure S4. Relative growth rate (RGR, % d^-1^) of *E. radiata* gametophytes among three separate populations for a 2050-centered future period (2040-2059) under climate scneario RCP4.5. The three populations are Coffs Harbour (low latitude, 28°S-31°S), Sydney (mid latitude, 31°S-34.5°S) and Tasmania (high latitude, 41.5°S-44°S). Monthly projections have been seasonally aggregated (Winter = June to August, Spring = September to November, Summer = December to February, Autumn = March to May).
