# Supplementary material for: Projecting kelp (Ecklonia radiata) gametophyte thermal adaptation and persistence under climate change
Source: Ann Bot. 2023 Sep 4;133(1):153–68. doi: 10.1093/aob/mcad132 (PMC10921825; doi:10.1093/aob/mcad132)
Supplement: mcad132_suppl_Supplementary_Table_S1 [file mcad132_suppl_supplementary_table_s1.docx]

Table S1. Details of GCMs (CMIP5) downscaled (0.05°) to support projections gametophyte survival and RGR off eastern Australia. The downscaled variable from each model include was sea surface temperature under RCP4.5 and 8.5 emissions scenarios

| Model | Institution | Native ocean resolution (°) |
| --- | --- | --- |
| ACCESS1.0 | CSIRO-BOM, Australia | 1.0×1.0 |
| CNRM-CM5 | CNRM-CERFACS, France | 1.0×0.8 |
| GFDL-ESM2M | NOAA, GFDL, USA | 1.0×1.0 |
| HadGEM2-CC | MOHC, UK | 1.0×1.0 |
| MIROC5 | JAMSTEC, Japan | 1.6×1.4 |
| CanESM2 | CCMA, Canada | 1.4×0.9 |
