# Supplementary material for: Projecting kelp (Ecklonia radiata) gametophyte thermal adaptation and persistence under climate change
Source: Ann Bot. 2023 Sep 4;133(1):153–68. doi: 10.1093/aob/mcad132 (PMC10921825; doi:10.1093/aob/mcad132)
Supplement: mcad132_suppl_Supplementary_Data [file mcad132_suppl_supplementary_data.docx]

Dear Prof. Hanley,

Thank you for overseeing the review of our manuscript. We are pleased that you and the reviewers perceived our manuscript favourably and welcome the opportunity to address and incorporate their comments and suggestions.

The main problem encountered by reviewers with our manuscript were 1) whether the data were reliable enough based on our methodology (the use of aquarium heaters), 2) the length of the discussion and 3) the statistical comparison of experiments that have been executed at different time points. We used aquarium heaters to establish temperature baths, but these fed into our aluminium temperature block, which kept remarkably stable temperatures. We have added extra data from temperature loggers to the supplementary material to further support this. In addition, we have cut two pages from our discussion, mainly the more descriptive parts. We were also happy to be alerted to the error we made in statistically comparing the separate temperature experiment for each population. We have amended the analysis so that each experiment is analysed separately. See the specific comments below for further explanation. All other minor suggestions have been incorporated into the revised manuscript and responded to in the below response table.

Thank you for your consideration,

Reina Veenhof

RESPONE TABLE:

| **Editorial comments** | **Response** |
| --- | --- |
| I certainly agree with the need to reduce length (currently 7 pages with 'Conclusions) to something under or around 4 pages. An obvious way to do this is (as Ref 2 suggests) reduce/omit the description of results in the first paragraph. Please focus on unpicking hypotheses and explanations. | ADDRESSED  We have reduced the length of the discussion. In particular we have reduced result descriptions in the first few paragraphs. This reduced the discussion from 7 pages to 5 pages. |
| I would also like to recommend you widen the impact of the MS by referring (briefly) to the review paper in the AoB 'Plants and Climate Change' Special Issue by Parmesan & Hanley (2015 doi:10.1093/aob/mcv169). On page 857 you can find a section that outlines the pivotal importance of understanding the regeneration stage in the context of understanding climate change. A quick mention of this theme and paper would be appropriate at around lines 90 and 380 of your manuscript. | ADDRESSED  Thank you, we have included this paper in the following lines:  L86: The gametophyte stage is considerably less researched than the sporophyte stage, but integral for kelp forest persistence (Veenhof *et al.*, 2022) as the stage through which regeneration occurs is pivotal in understanding species reaction to climate change (Parmesan and Hanley, 2015). |
| Problems (as addressed by Reviewer 1) focussed mainly on whether the data provided by the thermal experiments using aquarium heaters are a realistic approximation of 'real-world' scenarios and thus if the later range projections were founded on reliable data. | ADDRESSED  We have addressed this in our response to Reviewer 1. Briefly, through the use of an aluminium thermal block, the experimental temperatures were stable. We have added additional data to support this, which is available in the revised supplementary material as Figure S2. |
| **Reviewer 1** | **Response** |
| This highly original research provides an important addition to the field as usually projections only consider the persistence of adult sporophytes. | Thank you! |
| However, I have strong doubts regarding the accuracy of the data delivered from the thermal experiments as they were performed with aquarium heaters that usually do not keep the water temperature consistently stable. Can the authors demonstrate that the temperatures were stable throughout the days (and from the morning to the night) of the experiment? | ADDRESSED We tested our temperature block for stability with temperature loggers. Data from these loggers show a stable temperature both day and night (there is about 1°C variability). This is because, while we used aquarium heaters to heat the water in header tanks, the actual thermal range was established using an aluminium heat block, which kept stable temperatures within experimental treatments throughout the experiments. This can be seen in Fig. S1, where temperatures were measured at a random time between 9 am and 5 pm, every day. Additionally, we give the thermal curves of the temperature loggers as Fig. S2, as extra data on temperature stability. |
| It also seems that the thermal experiments were performed at different time periods for the distinct populations, so how statistical comparisons can be performed? | ADDRESSED  Thank you. This is an oversight on our part.  We have changed the statistical analysis from a two-way ANOVA including fixed factor treatment temperature and population, to three separate one-way ANOVAs with treatment temperature as a fixed factor. We have changed the methods and results to reflect these changes:  L217-219: We applied a generalized linear modelling (GLM) framework to assess for the effects of the fixed factor treatment temperature on the survival, relative growth rate and sex ratio of gametophytes of each population  L225-227: Significant effects of temperature were followed by pairwise comparison between sites for every temperature point using estimated marginal means and at a significance level of alpha = 0.05.  L292-303, L314-322 and L333-340 represent adapted text to describe the results of the adapted analyses. |
| Additionally, some methodological details are missing and need to be incorporated in the manuscript, so the readers easily understand how the experiments were performed. | See comments on the methods below |
| The discussion also needs to be improved, reducing the results description particularly in the first paragraphs, while incorporating more hypothesis and explanations. Some paragraphs in the discussion are too long, spreading for more than one page! | ADDRESSED  We have reduced the length of the discussion. In particular we have reduced result descriptions in the first few paragraphs. This reduced the discussion from 7 pages to 5 pages. |
| References are up to date and relevant and in a general way well utilised to provide background and justification for the work. The writing and language style is good. There were some errors in the figures that have been noted accordingly. | Thank you |
| Title  I think the title is quite misleading as it seems that the work is much broader, therefore I would recommend incorporating the studied kelp species (Ecklonia radiata) and location (eastern Australia). | CHANGED  We have changed the title to:  Projecting kelp (*Ecklonia radiata)* gametophyte thermal adaptation and persistence under climate change |
| Introduction  - Line 58: "... with losses documented in...". Losses of what, abundance, biomass, local extinction? Please specify. | ADDRESSED  Have specified on line 59-60:  ‘with losses of biomass, abundance and range as well as local extinctions documented in ..’ |
| - Line 62: The reference "Nicastro et al. 2013" is not appropriate as the work is focused on fucoides and not kelps. | CHANGED  Removed reference. |
| - Line 65: "...signifies a loss in overall biodiversity..." Overall marine biodiversity? | CHANGED  Added ‘marine’ on line 67 |
| - Line 66: "...fragmentation of kelp habitat reduces resilience of the remaining kelp forest, triggering further declines." Please explain better. | CHANGED  L66-69: ‘and fragmentation of kelp habitat reduces recruitment, triggering further kelp decline’ |
| - Line 71: "Range contractions are common along coastlines where is no suitable habitat for poleward range expansion." I do not totally agree with this statement as range contraction might happen anyway in the trailing edge even with suitable habitat and species expansion towards the poles. Please rephrase it for accuracy. | ADDRESSED  We agree and have changed it to:  L73-75: ‘Range contractions are common along coastlines affected by ocean warming and can be especially severe where there is no suitable habitat for poleward range expansion.’ |
| - Line 125: "...differential responses of thermally adapted gametophytes." Please be aware that the work performed in this study cannot prove local adaptation. I recommend rephrasing the sentence. | CHANGED  Have changed from ‘thermally adapted’ to ‘thermally adjusted’. See line 128. |
| Materials and Methods  Some minor issues in the text need to be addressed in order to clarify the methods used in the study, so the readers easily understand how the experiments were performed. | See below. |
| - Line 146: Gametophyte cultures were maintained at 19, 16 and 12ºC and the starting temperature of the thermal experiment should be the same (line 169), but in Figure S1 it says that the starting temperatures are: 20, 16 and 12ºC. Please revise the information. | CHANGED  We amended from 19 to 20°C. See line 149. |
| - Line 146: Please specify the type of equipment used for the different temperatures (climatic chambers, water baths?) and light intensity (LEDs, fluorescent lamps). Which daylength was used? | ADDRESSED  We have specified on lines 149-151:  ‘.. under an irradiation of 20 µmol/m^2^/s at a 12:12 cycle and 20˚C, 16°C and 12°C for Coffs Harbour, Sydney and Tasmania, respectively. Cultures were maintained in climatic chambers fitted with LED lamps. |
| - Line 149: For how long the gametophytes were growing before the start of the experiment? | ADDRESSED  See line 153:  ‘Coffs Harbour cultures were approximately 1 year old, Sydney cultures approximately 2 months and Tasmania cultures 2 years before use in this experiment.’ |
| - Line 159: I would not consider aquarium heaters as feasible equipment's to perform thermal experiments. | ADDRESSED  See comments above on stable temperatures and the use of an aluminium block. Though aquarium heaters were used to heat/cool header tanks, the water was pumped through an aluminium block that created stable temperatures throughout the experiment. |
| - Line 172: "One set of replicates (n=5) was kept outside of the temperature block at the respective holding temperatures for each population..." Indeed, 19, 16 and 12ºC should have been used as controls, i.e., these temperatures should have been included as experimental temperatures for all the populations... Were these replicates kept in exactly the same conditions than the others i.e., the same light intensity, daylength and light equipment? If not, the data from these replicates cannot be compared to the other temperatures. | ADDRESSED  These replicates served as a baseline, i.e. the conditions of each culture at the start of the experiment. They were measured at the start of the experiment, directly after being blended and settled. They have been maintained under the exact same holding conditions, and then one day of experimental conditions (which changed the light from 20 to 27 µm/m^2^/s) but otherwise were the same.  Changed line 177 to clarify:  ‘One set of replicates (n = 5) was kept outside of the temperature block at the respective holding temperatures of 19°C, 16°C and 12°C and under the experimental light conditions for each population and used as a baseline measurement to calculate survivorship and relative growth rate (see below)’ |
| - Line 172: "...used as a baseline measurement to calculate survivorship and relative growth rate." I do not understand what this means? Were the data of the other temperatures normalized regarding each starting temperature? Please explain better. | ADDRESSED  The exact calculations are given below, as indicated in the sentence. To clarify, we have changed it to (L179-181):  ‘Abundance, length and sex ratio of these replicates were used as a baseline to calculate survivorship and relative growth rate and sex ratio relative to the starting values for each population (see below).’ |
| - Line 176: "...temperatures ranging between 15.5ºC and 30ºC..." Please specify the temperatures used and be coherent how you present them through the manuscript. In this sentence is 15.5ºC and 30ºC, but in the figures is 15ºC and 30.5ºC. The temperatures are also not consistent between Figure S1 and the others. | CHANGED  Thank you, we have changed it to 15°C and 30.5°C.  Figure S1 contains the exact average measured temperatures, which we have rounded to 0.5 intervals for the ‘treatment temperatures’ used in the GLMs, for clarity. We have added this in the statistical methods to clarify.  L219-220: ‘Treatment temperatures as used in the GLM analysis were based on the measured averages (Fig. S1) but were rounded up at 0.5°C intervals.’ |
| - Line 177: The acclimation period lasted for 7 days for all the experimental temperatures and populations? Since the temperatures were changed at a rate of ~1.5ºC per day, how this was performed when for example the temperature was increased from 19ºC (holding temperature) to the experimental temperature of 20.5ºC? Was the temperature only increased on the last day of the acclimation? And how did you increase the temperature from 12ºC to 30.5ºC in only 7 days at a rate of ~1.5ºC per day. This information is unclear and needs to be added into the text. From Figure S1, the temperature changes do not seem to be performed at a rate of ~1.5ºC per day since the change is sometimes very sharp or very smooth... | ADDRESSED  The two water baths were each changed from 1-2.5 °C daily over a period of 7 days for each experiment, depending on the starting temperature.  We used the ~ 1.5 as a general indication, but we agree that this is not clear. We therefore changed it to 1-2.5.  The water baths were changed until the final temperatures on each extreme of the block were reached. This means that, the middle temperature ranges were reached at a slower rate than the extreme temperatures, as both ends got increasingly hotter/cooler. This is clearly visible in Figure S1, which is why we included it.  See line 182-185:  Temperatures in the water baths were altered at a rate of 1˚C -2.5 each day (24-hour period) depending on the starting temperature, until the final temperatures ranging between 15˚C and 30.5˚C were reached across the thermal gradient block (Fig. S1). This acclimation period lasted for 7 days for each experiment. |
| Figure S1: In the mid latitude population, the different experimental temperatures did not start from 16ºC?? | CHANGED  Thank you for catching that, the graph cuts off the first day. We have amended Figure S1 to correct this. |
| - Line 178: "Temperatures were checked daily..." I do not think that a daily temperature check is enough to prove stability. Was the temperature checked always at the same hour of the day? The temperature might fluctuate a lot when the lights are turn on or off (i.e., when there is a change from day to night and vice versa), particularly with fluorescent lamps. | ADDRESSED  The temperature was checked at random times each day between 9am and 5pm. In addition, we tested stability throughout day and night with a temperature logger, as an extra control to make sure the temperature block was stable. We did this separate from the experiments, as we could not add loggers to the beakers during an experiment as it would disturb the gametophyte cultures. This data is now included as Figure S2 as additional proof that the temperature block was sufficiently stable.  Line187-190: ‘In addition, a wireless high resolution temperature logger was used to measure the lowest (15 °C) and highest (30.5°C) temperature treatments every 10 minutes for 4 days, to ensure stability in the temperature block (see Fig. S2). |
| - Line 179: It seems that the thermal experiments with the different populations were performed in distinct dates. How can the data from different populations be statistically compared? | ADDRESSED  See comments above.  We have changed the analysis to three one-way ANOVAS:  L217-219: ‘We applied a generalized linear modelling (GLM) framework to assess for the effects of the fixed factor treatment temperature on the survival, relative growth rate and sex ratio of gametophytes of each population.’  L225-227: ‘Significant effects of temperature were followed by pairwise comparison between sites for every temperature point using estimated marginal means.’ |
| - Line 182: "After 14 days, the response of gametophytes to the thermal gradient was recorded." At day 0, also?? | CHANGED  L192: ‘At the start of the experiment (baseline measurement) and after 14 days, ..’ |
| - Line 187: "The number of individuals, males, females and juvenile sporophytes..." What do you consider individuals? Are not the female and male gametophytes? | CHANGED:  L197-199: Clarified to: ‘the number of individuals (both male and female), as well as number of males, females and juvenile sporophytes per FOV (three for each replicate) was counted’ |
| - Line 197: "...represent maximum branch length..." Do you mean gametophyte length? It was easy to measure gametophyte length? Gametophytes might not grow as a straight line but be curved or like a cluster, how did you perform the measurements in these cases? | ADDRESSED  Maximum length was measured as the length of a branch (if curved, it was measured in segments using the segment tool in ImageJ to get a close approximation) or the diameter of the cluster if it grew in a cluster.  We changed ‘maximum branch length’ to ‘gametophyte length’, see line 196 |
| Results  - Line 283: I think that is a bit strange that the low latitude population presented gametophyte survival percentages lower than 100% in all the temperature treatments. This might indicate that the gametophytes were not healthy in the beginning of the experiment... And how do you explain that only ~30% of the gametophytes survived at temperatures ranging 15ºC-20.5ºC, but the ones surviving were growing very well? | ADDRESSED  The higher mortality in the low latitude population could be due to less healthy cultures, or it could be an effect of being situated closer to the range edge, where there is a different trade-off for survival and growth.  This is discussed in the discussion:  L449-460: ‘While the projected survival for the low-latitude population ranged between 30-70%, their projected RGRs were the highest among populations. Conversely, high-latitude populations had the lowest projected RGRs, down to 0 in autumn. This may indicate separate trade-offs between survival and growth for lower and higher latitude populations. Low survival but high RGRs in warmer temperatures cause fewer, but larger gametophytes in the low-latitude populations, and high survival but low RGRs in warmer temperatures cause a greater number of smaller gametophytes for the high-latitude population. A similar trade-off to that of the low latitude populations under increased temperatures (lower survival but increased growth) has been observed for E. radiata gametophytes across Western Australia (Mohring et al., 2013). Larger female gametophytes produce more eggs per female (Muñoz et al., 2004, Camus et al., 2021), and this may compensate for reduced fertility resulting from higher mortality and lowered fecundity in the low latitude population (Camus et al., 2021).’ |
| - Line 319: "For high latitude gametophytes, ... declining to 0% d-1 at 25ºC...". The RGR of high latitude gametophytes is already 0 at 20.5ºC (shown in Figure 2A)? | CHANGED  Correct, thank you for pointing that out. We have changed it to:  L320: ‘High latitude had a significantly lower RGR from 20.5°C onward (<1 % d-1, p<0.05, Fig. 2A). |
| - Line 325: I would consider more accurate to say that the male gametophytes are decreasing i.e., more male gametophytes are dying compared to female gametophytes due to the high mortality observed with the temperature increase. There was not an increase of the number of female gametophytes (i.e., no more female gametophytes developed). | CHANGED  We agree, changed it to:  L334-336: ’Pairwise comparison showed a significant decrease in male gametophytes in the low latitude population at 29°C and 30.5°C compared to lower temperatures (p<0.05, Fig 3A). Mid latitude gametophytes stayed at an approximate equal sex ratio (Fig. 3A).’ |
| - Line 337: "Projections of gametophyte survival and RGB varied..." Do you mean RGR instead of RGB? | CHANGED  Yes, corrected (L350). |
| - Line 339: Figure 6 is mentioned in the text before Figure 4 and 5, therefore the authors should change the order, i.e., Figure 6 should be Figure 4. | CHANGED  Indeed, we have changed the figure order. |
| - RGR and survival results: Comparing the survival and the RGR results the most striking finding was that the observed mortality in the low latitude gametophytes was not supported by the RGR results as the gametophytes grew very well. | ADDRESSED  We address this in the discussion, see L449-460 and comments above on the same result. |
| - Figures 1A, 2A and 3A: Please include the statistical letters from the interaction between temperature and population in the figures, so it is easier for the readers to follow the results text. | CHANGED  We have added statistical letters referring to posthoc tests of each one-way ANOVA to the figures. Revised Figure 1, 2, 3 are included. |
| - Figure 2A: The bars from 27ºC onwards are wider than the others. Please revise this. | CHANGED  Have changed the bar width in Figure 2A |
| - Figure 3A: The thick red lines included above the bars are representing what? Please include information in the Figure legend. The bars from 25.5ºC onwards are wider than the other. Please revise this. | CHANGED  Have changed bar width in Figure 3A.  Have added explanation on tick red lines in Figure 3A legend.  ‘The red dotted line denotes a 0.5 sex ratio, where there is an equal number of males and females’ |
| - Figure 4 and 5: Please include in the figure legend what is A, B and C. | CHANGED  Added in figure legend (now 5 and 6):  L857 and L863:‘The three populations are A) low latitude (Coffs Harbour, 28°S-31°S), B) mid latitude (Sydney, 31°S-34.5°S) and C) high latitude (Tasmania, 41.5°S-44°S).’ |
| - Figure 4, 5 and 6: I recommend incorporating low, mid and high latitude in the y axis of each panel of graphs so the reader can easily associate the graphs to the population. | ADDRESSED  With the addition of the populations in the Figure legend (see comment above), each panel should be easy to understand. |
| Discussion  In my opinion the discussion needs to be improved, reducing the results description particularly in the first paragraphs, while incorporating more hypothesis and explanations. If genetically distinct populations were used why the different thermal tolerances observed where not discussed taking into consideration the genetic diversity. | ADDRESSED  We agree and we have reduced the length of the discussion. In particular, we have reduced result descriptions in the first few paragraphs. This reduced the discussion from 7 pages to 5 pages.  We based the populations on genetic data that has not been published yet, and so the genetic data is not explicitely discussed in the discussion. |
| - Line 385: "...which was ~2-3ºC above their current in situ thermal maxima" Can the authors please include information in the manuscript regarding the current in situ thermal maxima for each population? | ADDRESSED  We have provided this information in ensuing paragraphs (see line 402 – line 407). |
| - Line 386-391: These two sentences are just a repetition of the results. I recommend the authors to reduce the description of the results and integrate hypothesis, explanations i.e., discuss the results. | CHANGED  We have removed this paragraph. |
| - Line 396: "... indicating E. radiata gametophyte populations are well adapted for present-day temperatures and future ocean warming." Which populations are well adapted? All populations or only the low latitude population presenting high survival? Not clear. | ADDRESSED  This paragraph has been removed. |
| - Line 399, 402, 408: "...adaptation to local temperatures..." Local adaptation can be detected when local populations perform better than foreign ones in reciprocal transplant experiments, therefore this study cannot prove the existence of local adaptation as well as most of the studies cited, they show the existence of plasticity and might only suggest adaptation to local temperatures. Please tone down the sentences regarding adaptation. | ADDRESSED  Changed wording to:  L397: ‘which can suggest adaptation to local temperatures.’  L400: ‘We found evidence thermal plasticity among populations of *E. radiata* gametophytes, which was related to local temperatures.’  L407: ‘Thermal plasticity in accordance with local temperatures among geographically distinct populations of gametophytes..’ |
| - Line 404: "Survival was higher for the low latitude gametophytes in the warmer temperature treatments (22.5-30.5ºC)..." From Figure 1A is clear that survival is near zero above 27ºC, therefore consider changing to "...(22.5-27ºC)..." Additionally, replace "...this populations..." by "...this population...". | CHANGED  L403: Changed from 30.5°C to 27°C.  L403: Changed from ‘population’ to ‘populations’ |
| - Line 441: Please consider that decreased survival of female gametophytes compared to males is also observed in several kelp species exposed to high temperatures (Bolton and Lüning 1982; tom Dieck 1993; Komazawa et al. 2015; Martins et al. 2020). I would include in the discussion geographic parthenogenesis as a hypothesis to explain the sex ration favouring females in the two range-edge populations. | ADDRESSED  We have incorporated suggested information on the decreased survival of females in other populations. We have added:  L437-442: ‘Increased survival of females under increased temperature at range edges has also been observed in *Lessonia nigrescens* (Oppliger *et al.*, 2011) and *Macrocystis pyrifera* (Rodriguez *et al.*, 2019). This contrasts with other, non-range-edge populations, where survival of males is higher under temperature stress (e.g. Bolton and Lüning, 1982, Komazawa *et al.*, 2015, Martins *et al.*, 2020). An increased number of females at lower latitudes may help increase fecundity, as number and size ..’  We do not think parthenogenesis explains the increased number of females, as sex ratios were near equal at the start of the experiment. However, increased number of female gametophytes may lead to parthenogenesis occurring, which is more common in range edge populations. We have added:  L444-L447: ‘An increased number of females may also result in parthenogenesis, asexual reproduction through female gametophytes, which can occur in range edges in response to marginal conditions (Oppliger *et al.*, 2014).’ |
| - Line 450: Consider replacing "...of separate populations..." by "...of different populations..." | CHANGED |
| - Line 481: "The mid-latitude population was identified as the most vulnerable, with a projected 30% decrease in survival." It is a bit surprising that a central population is the most vulnerable one, we always expect that trailing edge will more affected. Can the authors provide some more explanations to justify the vulnerability? | ADDRESSED  This is likely because the thermal tolerance of the mid-latitude population is lower than that of the low-latitude population, but some of it’s northern extend will experience the same increase in temperature in a 2050 scenario.  We have added:  L476-L483: ‘This is due to a relatively lower thermal tolerance of that population compared to the low-latitude population, while some parts of the mid-latitude population would experience temperature increases similar to the low-latitude population. However, the low-latitude population had a projected 0% change in survival, and high connectivity among *E. radiata* populations along the eastern Australia (Coleman *et al.,* 2011a, Coleman *et al.,* 2011b, Coleman *et al.,* 2017) may result in more thermally tolerant gametophytes migrating to higher latitudes, potentially increasing projected survival rates of the mid-latitude population.’ |
| - Line 490: "...persisting through temperature extremes of up to 27-30ºC..." This is only true for low latitude gametophytes. | CHANGED  L487: added ‘in the lower latitudes’. |
| Conclusion  - Line 503: "...E. radiata gametophytes are adapted to local temperatures..." Again, this study cannot prove the existence of local adaptation, only intraspecific thermal plasticity. Please rephrased it. | CHANGED  L501: Rephrased to: ‘have thermal tolerances in accordance with their local temperatures across 13 degrees’ |
| **Reviewer 2** | **Response** |
| This was a joy to review, thank you. Very tidy study and elegantly designed experiment that adds much to the existing literature. All this dovetails nicely into the modeling of the data to inform future response and state of E. radiata populations to thermal increase. Job well done. A crucial missing piece of the modeling puzzle. | Thank you! |
